# Intentional synchronisation affects automatic imitation and source memory

**DOI:** 10.1038/s41598-020-79796-9

**Published:** 2021-01-12

**Authors:** Liam Cross, Gray Atherton, Natalie Sebanz

**Affiliations:** 1 Department of Psychology, Edge Hill University, Liverpool, L39 4QP UK; 2 Department of Cognitive Science, Central European University, Budapest, Hungary

**Keywords:** Psychology, Human behaviour

## Abstract

Acting in synchrony is a fundamental part of many social interactions and can have pro-social consequences. Explanations for this relationship were investigated here using implicit measures of imitation (automatic imitation task) and memory (preference overlap task). In Study 1, participants performed an intentional synchronisation task where they moved sliders in or out of time with another person while a third person observed. Those who had moved in synchrony showed a stronger tendency to imitate their partner’s actions than those who had moved in a non-synchronous way. Similarly, coordinated partners were also more likely to share object preferences. Results also showed that rather than memory blurring between co-actors, participants had improved memories for the self. Study 2 exchanged intentional for incidental coordination (coordinating with a synchronous metronome). None of the findings from Study 1 replicated when synchronisation was incidental rather than intentional, suggesting that having a shared goal may be critical for triggering effects of synchronisation on imitation tendencies and memory. Together these findings favour explanations related to changes in social categorisation over representational overlap between co-actors.

## Introduction

We rhythmically coordinate with each other throughout our lives, from falling into step as we walk next to each other to dancing, singing, and playing music together^1,2^. Generally, rhythmic coordination occurs when the movements of co-acting individuals follow specific patterns of relative timing between them^3^. A common form of rhythmic coordination is (in-phase) synchrony, where people perform the same movements at the same time as one another^4^, either because they have a shared goal to do so, or because they happen to have fallen into a synchronous pattern.

Moving in synchrony with others fosters many social consequences. For instance, we are more likely to help, cooperate, conform with and obey those with whom we have acted in synchrony^5–7^. The relationship between synchrony and its socio-emotional consequences is demonstrated in a rapidly growing body of empirical work, and various explanations for this relationship have been purported (for recent reviews see^8–10^).

Some suggest that acting in synchrony leads to a positive affect and generalised pro-sociality^11^. That is, moving in time leads people to feel good and act more prosocially or positively towards people in general, including towards individuals with whom they have not coordinated. However, while some work suggests that synchrony leads to generalised pro-sociality^11,12^, other work does not support this conclusion^13–17^. Indeed, see^8,15^ for an alternative explanation for the findings. There is also little evidence for positive affect underlying the relationship between synchronous movement performance and generalised pro-sociality^7,8^.

Other explanations revolve around synchrony leading to an increase in representational overlap between self and other, which can be reflected in co-actors feeling close to each other. Some go as far as to suggest that we essentially begin to represent our co-actors as a part of, or an extension of, ourselves^18^. Others suggest that synchrony makes us see those we coordinate with not as part of or an extension of ourselves, but as part of the same social group as ourselves^8^. These explanations do not suggest that coordination’s effects will extend beyond those whom we have synchronised with to include other people (although there is some evidence^14,19^ in line with the group categorisation explanation that the effects of synchrony may extend to affiliates or wider group members of our co-actor).

There is mixed evidence as to whether moving in synchrony leads to changes in representational overlap between the individuals involved. While some work finds more reported overlap and affiliation between co-actors following synchronous coordination^7,20^, others have failed to replicate these findings^12,21–23^. All of this work has relied on explicit self-report measures of similarity, closeness and connectedness between co-actors or pictorial ratings of overlap using the Inclusion of Other in Self scale (IoS)^24^. Similarly, there is also some evidence that seeing oneself and one's partner less as an individual and more as a member of a group may mediate the relationship between synchrony and pro-sociality^25^.

These measures suffer from issues relating to social desirability and experimenter effects^26^ as well as potential limitations regarding introspective ability^27^. More recently, work has started exploring effects of synchrony using more implicit measures relying on reaction times and error rates^19^ and memory tests^28–30^. To contribute towards a better understanding of the mechanisms whereby acting in synchrony affects social relationships and social behavior, the present study explored the effects of synchrony using implicit measures that have been found to be influenced by group categorisation and self-other overlap.

The automatic imitation task (AIT) (specifically the forced-choice RT task version used here) assesses the degree to which an individual is influenced by the task-irrelevant movements of another individual (which may be either congruent or incongruent with the movements to be performed) while responding to a target cue. In one of the original studies of automatic imitation, Stürmer^31^ had participants respond to cues instructing them to either open or close their hands in response to one of either two colours overlaid on a video showing a hand opening or closing. Participants were faster when the video showed a task-congruent hand (i.e. an open hand when the color cue matched the open hand instruction) than when the video was incongruent with the color cue (i.e. an open hand when there was a color cue for a closed hand instruction). Essentially, participants, though not instructed to attend to the hand cues, were influenced by the other hand’s movement, revealing an underlying tendency to automatically imitate the observed hand’s actions.

While a large body of work has provided evidence for automatic imitation, the specific processes involved have been debated, and it is assumed that effects in the AIT reflect a composite of spatial and imitative compatibility effects^32–34^. Importantly, research suggests that social context influences the automatic imitation effect^35^. For example, pro-social priming has been shown to increase the tendency for automatic imitation^36^. Stronger imitation of in-group members has also been reported. Gleibs^37^ showed that in non-competitive situations, participants showed greater automatic imitation of individuals who were part of their own social group, compared to those who were not. Based on these reported social modulations, we explored whether participants would have a stronger tendency to imitate the hand movements of someone they had moved in synchrony with compared to someone with whom they had moved out of time. If this proved to be the case, we also were interested in exploring whether this effect extended to someone who merely observed but didn’t participate in the movement task. Accounts postulating that synchrony leads to generalised pro-sociality predict that synchrony would lead to a stronger tendency to imitate observers as well as co-actors, provided that pro-sociality increases imitative tendencies. By contrast, accounts postulating that synchrony affects self-other overlap and/or group categorisation predict that if synchrony affects imitative tendencies, this should be restricted to the co-actor who participated in the synchronous movements.

To better understand the role of social categorisation and self-other overlap, we additionally investigated the effects of synchrony on memory performance in the preference overlap task (POT). In this task, individuals are shown pairs of objects and are asked to indicate either which of the two items they prefer, or which object they believe two other people would prefer. In a surprise memory test, they are later asked to recall which object they had chosen and for whom they had made this choice. This memory task makes it possible to assess source confusion towards one of the given agents (that is, incorrectly recalling an item as having been chosen for agent A when it was in fact selected for agent B). Studies using the POT^38^ have shown that participants will be more likely to confuse their own choices with choices they made for someone they perceive to be similar (typically a friend or family member) versus someone perceived as more dissimilar (typically a stranger). For instance, a person is more likely to confuse whether an item was chosen for ’ ‘self’ or ‘mother,’ and to be less confused about whether the item was chosen for ‘self ‘or ‘stranger’^39^.

The memory test of the POT task can be used to assess if having acted in synchrony increases source confusion between self and co-actor, as predicted by the overlap explanation. If indeed synchrony leads us to represent others as a part of our self, one would expect more significant source confusion between choices referring to the self and those referring to a co-actor with whom the participant had moved in synchrony. On the other hand, if synchrony affects group categorisation, one would not necessarily expect synchrony to lead to greater source confusion with co-actors.

The POT also tests a second assumption, that a person will use the self as a proxy for predicting the preferences of similar versus dissimilar others, in line with egocentric biases in mental state attribution (e.g.,^40^). Research suggests that the relation between similarity in preferences and perceived affiliation is bi-directional. That is, we are more affiliated with people who like the things we like, and we predict that we will like those who like what we like more than those who do not share these preferences^41^. Thus, the POT uses preference overlap to measure the degree to which we assume another is ‘like us’ (by assessing if we choose the same objects for another agent as we would for ourselves).

Accounts postulating that synchrony affects self-other overlap and/or group categorisation predict greater preference overlap with the synchronous partner in the POT. While self-other overlap explanations would also predict greater source confusion with the synchronous co-actors, explanations based on group categorisation would not predict greater source confusion.

In the present study we instructed pairs of individuals to either move in in-phase synchrony (Synchronous Condition) or to perform the same task but at different rhythms (Non-synchronous Condition), while another person (the Experimenter) observed but did not partake in the task. Following this, participants’ tendency to imitate actions of their Partner and the Experimenter in a task where these actions should be ignored was measured, along with their affiliation towards them, and their (memory of) preference choices made for these individuals.

## Study 1

### Method

#### Participants

Fifty-six participants were recruited from the Central European University participant pool (35 females, 20 males, 1 other; M_age_ = 23.34 yr, SD_age_ = 3.79). All participants were right-handed, naive to the aims of the study, and compensated with 1500 HUF (approx $7). We based our sample size on Gleibs^37^, which suggested that a sample size of 18 per cell was sufficient to achieve 80% power based on effect sizes in the IAT literature^36,42–44^. Given that Gleibs^37^ found a slightly smaller effect size than previous literature, which is also in line with effect sizes seen in behavioural measures of the pro-social effects of synchrony^9^, we followed their recommendation of achieving > 25 people per cell. The experiment was approved by the (EPKEB) United Ethical Review Board for Research in Psychology and took approximately 40 min to complete. All research was performed in accordance with relevant guidelines/regulations, and informed consent was obtained from all participants.

#### Design and procedure

The study employed an experimental design with one between-subjects factor: Joint movement, having two levels, Synchronous versus Non-Synchronous. The study was conducted in pairs, and each participant was unfamiliar with their experimental partner. Upon entering the lab, participants were introduced to each other by name and informed that they would be participating in an experiment investigating how people perform different kinds of movement tasks.

The participants and the Experimenter were then assigned colored gloves, which they wore for the duration of the experiment. The use of the gloves was to ensure that they would identify hands seen in videos during the AIT task as belonging either to the Experimenter or their movement partner. To blind participants to the purpose of the task, they were informed that the motion tracking equipment used to measure movement could cause static excess energy, and everyone would need to wear gloves while in the lab. The Experimenter wore blue, and the participants wore white and gray gloves, respectively.

After everyone had put on their gloves, participants were told that for one of the tasks they were doing later they would see a video of either the experimenter’s, their own or their partner’s hand performing an action. These videos were then recorded, starting with the experimenter. Everyone sat at a table covered with a black cloth, held their hand up at an angle of 90 degrees, pointing towards the camera. After a count down from 3 s, they dropped their index finger of their right hand and kept it pressed down for a further 2 s. They then recorded the second video, this time dropping their middle finger. After the experimenter, each of the participants recorded these stimuli. This was done in order for participants to believe the videos they saw in the AIT task showed their partner and the experimenter.

Participants first completed an explicit measure of affiliation, followed by either a Coordinated or Uncoordinated movement task, followed by either the AIT or the POT (the order of these tasks was counterbalanced between pairs). After this, a second, identical version of the movement task was performed, followed by whichever of the AIT/POT tasks remained. Participants finally answered some questions regarding task perception, difficulty, and enjoyment, along with a second copy of the affiliation measure. All responses were recorded individually and privately on a computer, and no discussion between participants during the experiment was allowed. Participants were then funnel- debriefed and assessed for knowledge of the study aims and hypothesis.

#### Tasks and measures

##### Movement task

**Figure 1:**
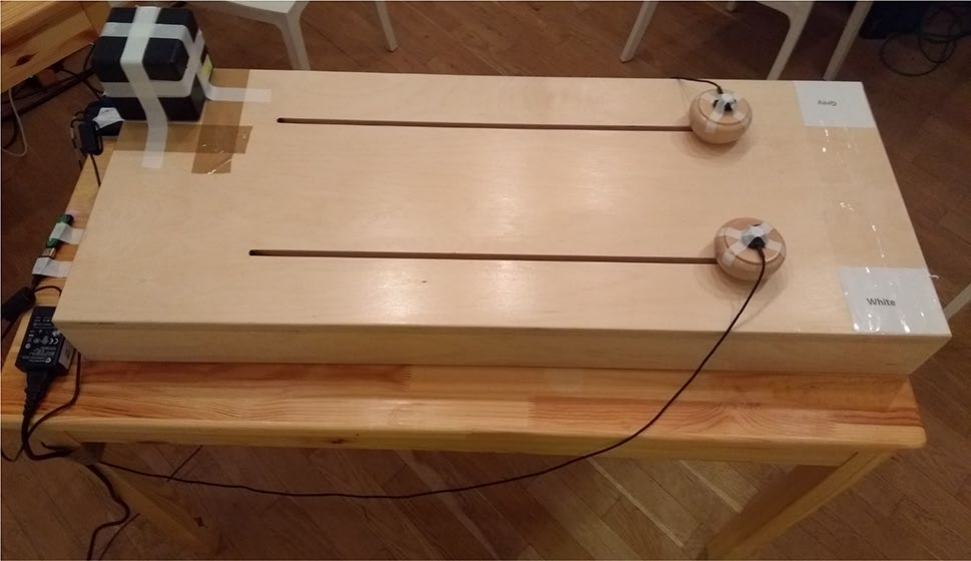
The slider board.

The movement task involved participants each moving one of two sliders on a slider board^45^. The board was a rectangle measuring 80 × 40x10cm and was made entirely of wood (see Fig. 1 for reference). Sliders were 8 cm (diameter) and 4 cm (height) positioned 20 cm apart and each slider moved along a 60 × 1 cm track. We recorded movement data using a Polhemus G4 motion tracker and attaching sensors on top of the sliders (sampling rate of 120 Hz, using custom tool kit on Matlab^46^. We created coordination scores for each trial separately by taking the squared difference between each slider’s position (in cm) every 100 ms, after excluding the first 20 s of each trial in order to exclude the part of the task where the metronomes had paced participants. We then took the square root of the average of the squared difference score for each trial. The resulting score is the average distance each slider was from the other over the trial in cm.

Participants in both conditions were asked to hold the slider with both hands and move it from side to side, using the full range of motion without hitting the endpoints. They were asked to move their slider at a steady, continuous pace to arrive at each endpoint in time with an auditory metronome which they heard for the first 20 s of the task to initially pace their movements.

In the Synchronous condition, participants were asked to work together to reach the endpoints of the board together at the same time as the beep and heard the same metronome (37 BPM) out loud. In the Non-synchronous condition, each person was asked to move to reach the endpoints of the board at the same time as the beep, and they heard different metronomes, 32 vs 50 BPM through headphones. Therefore, in the Synchronous condition participants were moving roughly in-phase (0 degrees) relative to each other, while in the Non-synchronous condition, there should be no perceivable phase relationship. In the Synchronous condition, participants also had a shared goal to coordinate, whereas in the Non-synchronous condition, they had different goals.

Because in-phase coordination, and to a lesser degree anti-phase coordination, are known to be strong attractor states to which people will drift when performing movement tasks^4,47^, in the Non-synchronous condition one half of the slider board was occluded from each participant's view using a plastic screen to keep participants from drifting into coordinated movements. Participants could still see each other’s head and shoulders but could not see their partner’s arm movements. Participants were first given two practice trials of 120 s to familiarise themselves with the required pace and movements. During these practice trials, the Experimenter corrected any errors. After this initial practice period, each of the three rounds of the movement task lasted for 180 s.

##### Affiliation

This measure consisted of five self-report questions taken from previous work^48^. These questions measured how close, connected, and similar participants felt to each other, how much they felt on the same team, and how much they would like to see each other again. Participants recorded their responses to each of these questions on a continuum ranging from ‘not at all' to ‘very much so' (scored from 1 to 100). This response scale was used to make it more likely to detect any changes after the movement manipulation and has been successfully used in a similar context^48,49^. A composite affiliation change score (after–before) was computed for affiliation by subtracting the initial score from the subsequent score for each item and taking an average across the five items.

##### Automatic imitation task

For the AIT, participants had to respond to a symbol presented on the screen with a button press, using a customised PsychoPy program. Participants were instructed to hold their right hand over a button box with their index finger over the X button, and their middle finger over the O button. If participants saw an X, they had to press the X button using their index finger, and if O the O button using their middle finger. They were instructed to do this both as quickly and as accurately as possible. Before seeing the relevant X or O, a static image of a gloved hand in a neutral position was shown. The hand wore either a white or gray glove, representing the Partner’s hand, or a blue glove, representing the Experimenter’s hand. This static image was presented for either 300, 600 or 900 ms. Of the 48 blue/white/gray glove trials, 16 showed the static picture for 300 ms before the cue appeared, 16 for 600 ms, and 16 for 900 ms. The order of the trial types was randomised. Following the static image of varying length, the cue was then presented alongside a now animated video of the hand performing either a congruent or an incongruent movement (either an index or a middle finger drop depending on the trial type). The static images were presented for a variable length of time in line with recommendations^50^, to keep participants from predicting the movement and from responding early.

**Figure 2:**
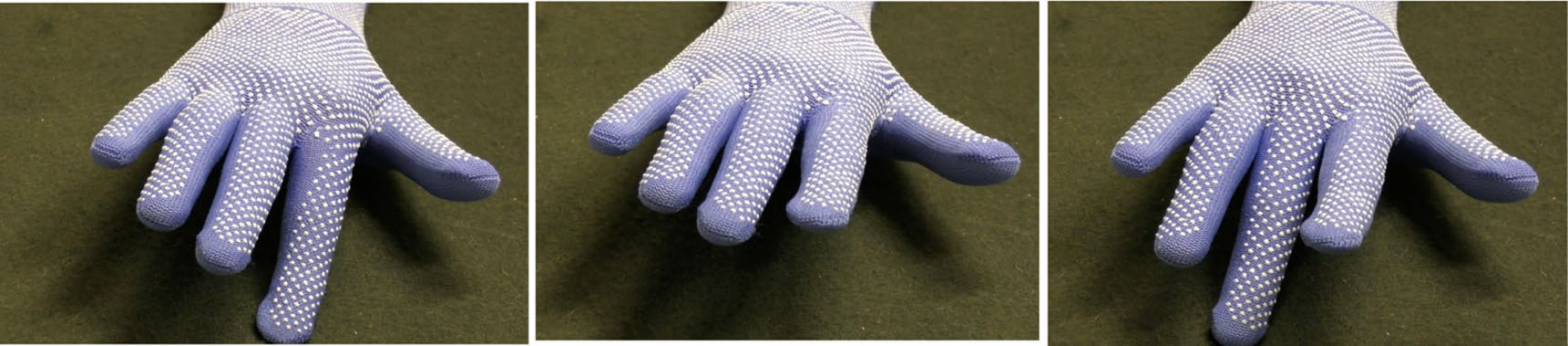
Example frames from the videos used in the automatic imitation task.

Twenty-four of the trials were congruent, meaning that the movement in the video matched the movement needed to execute the cued button press correctly. The other 24 were incongruent, meaning a mismatch between stimuli (for instance, a video showing the hand performing the movement pertaining to an X button press even though an O symbol was shown on the screen). Actual videos used were not the videos recorded at the start of the experiment but standardised videos (see Fig. 2 for examples of the videos used). This version of the task was based on that used by previous studies^37,51^.

Before the experimental trials, participants each performed eight practice trials to familiarise themselves with the task. In the practice task, they saw a hand wearing a pink glove (which did not correspond to either self, partner or experimenter’s hand) making a congruent and incongruent X and O button press (4 practice trials in total).

The Inverse Efficiency score (IE) was calculated separately for imitation towards ‘Experimenter's' and 'Partner's' hand. The Inverse Efficiency was chosen over other methods as it incorporates both error rates and response times, therefore giving one metric for imitation tendency per observed hand (Experimenter/Partner). This was calculated in line with recommendations^51^. To calculate the Inverse Efficiency (IE), any RTs belonging to incorrect trials and any RTs < 100 ms or > 1000 ms, were excluded. The error rate (ER) was then calculated for congruent and incongruent trials. Response times were divided by the proportion of correct responses (1- ER) to form separate IE scores for in/congruent trials, respectively. The overall imitation effect (IME) is the IE of the incongruent trials minus the IE of the congruent trials.

##### Preference overlap task

**Figure 3:**
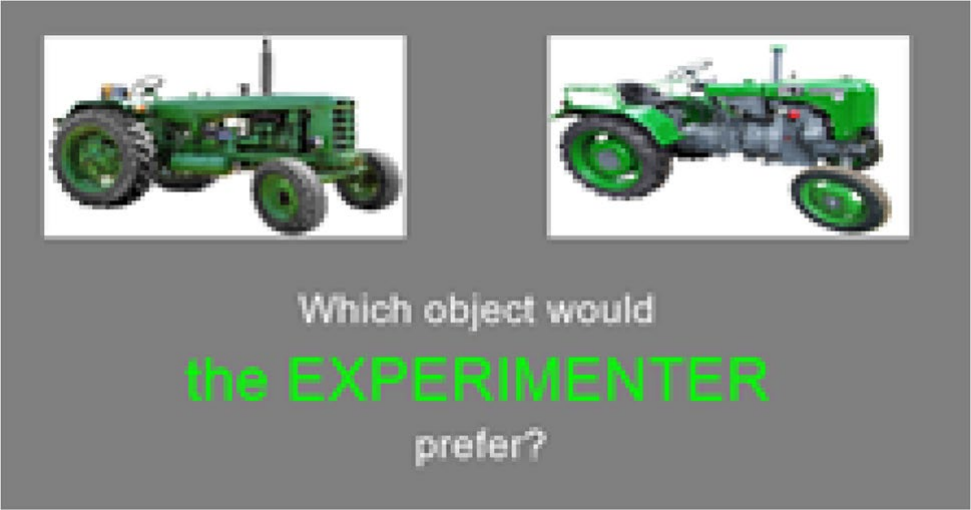
Example items from the preference overlap task.

For the POT task participants were presented with 48 different pairs of objects on a white background using a customised PsychoPy program. This task was based on previous studies^38^. Items within the object pairs were very similar to one another but not identical, such as the two tractors shown in Fig. 3.

Participants were asked to indicate which item either they would prefer, their  movement Partner would prefer, or the Experimenter would prefer, by clicking on the relevant image. Who an object pair was presented for (Self, Partner or Experimenter), and the order of presentation were all randomised. There were 16 trials each for Self, Partner, and Experimenter. Once participants had finished this task, they took a three-minute break before moving on to the next task. They were then presented with a surprise memory task. For all of the object pairs they had previously encountered they were asked to recall a) which object was previously chosen and b) for whom it was chosen. The order of objects presented in the memory task was randomised. Finally, participants were presented with all of the object pairs they had first selected for the Experimenter and their Partner and asked which one they themselves would prefer.

Preference imitation measures how many items that had previously been chosen for either the Partner or the Experimenter were then chosen for the Self. A preference overlap score was calculated by computing the percent of items first chosen for either the Partner or the Experimenter that was later chosen for the Self. Blur scores indicate the degree to which participants blurred choices for one individual with another individual when recalling for whom an item had been selected. Three blur scores were computed which measured blurring between Self-Partner, Self-Experimenter and Experimenter-Partner, by tallying the number of misattributions between the two relevant individuals (for example, how many times a participant incorrectly listed an item as having been chosen for the Self rather than Partner and vice versa). This was then transformed into a percentage which reflected the percentage of items that were misattributed to a given individual rather than the correct individual.

#### Analysis

Data was analysed throughout using a frequentist approach. Where data violated parametric assumptions, non-parametric alternatives were used, and no data was removed. In instances were null findings were close to the 0.05 cutoff point, Bayesian analysis was used to supplement frequentist tests. Bayesian analysis was done in JASP using default priors.

## Results

### Coordination

We first analysed the movement data gathered from the joint movement task in order to ensure people had performed the task adequately. Participants in the Synchronous condition did coordinate their movements more tightly than those in the Non-synchronous condition in both of the trials of the movement task, Trial 1 (U = 784.0, p < 0.001, r = 0.86) and Trial 2 (U = 756.0, p < 0.001, r = 0.86), see Table 1 for descriptive statistics. This confirmed that groups were clearly differentiated with regards to the amount of synchronous movements performed, allowing us to interpret the below results.

**Table 1:** Descriptive statistics for the joint movement task.

	Mean	SD	Median	Range
**Movement trial 1**				
Synchronous	2.31	0.61	2.18	1.64–4.09
Non-synchronous	26.97	2.83	26.76	22.4–33.54
**Movement trial 1**				
Synchronous	2.18	0.54	2.10	1.57–3.72
Non-synchronous	25.70	3.39	26.65	16.43–29.31

#### Affiliation

Scale analysis confirmed that the five affiliation items could be reliably combined into a single measure (Cronbach's alpha = 0.878). An independent samples t-test showed that those in the Synchronous condition (m = 5.429, SD = 10.32) reported significantly greater positive changes in affiliation than those in the Non-synchronous condition (m =  − 0.121, SD = 9.57) (t(54) = 2.087, p = 0.042, d = 0.55). A Bayesian t-test confirmed that the research hypothesis (that the Synchronous condition had greater affiliation scores than the Non-synchronous condition) was better supported (BF10 = 3.10) than the null (BF01 = 0.32). One sample t-tests also confirmed that only the change scores of the Synchronous condition significantly differed from 0 (t(27) = 2.78, p = 0.01, d = 0.52), while those of the Non-synchronous condition did not (t(27) = 0.067, p = 0.95, d = 0.012).

#### Automatic imitation

Data from one pair was not recorded due to program failure. For imitation tendency of the Partner's hand, those in the Synchronous condition showed greater automatic imitation than those in the Non-synchronous condition (u = 18,050, p = 0.01, r = 0.35). However, for imitation tendency of the Experimenter's hand, there was no significant difference between the Synchronous and Non-synchronous conditions (u = 266.5, p = 0.371, r = 0.12); please see Table 2 for descriptive statistics. Mean RTs and error rates reported separately for in/congruent trials can be found in the supplementary materials. This shows that after synchronizing their movements with a task partner, participants were more likely to imitate the Partner automatically, but synchronization did not affect imitation of the Experimenter.

**Table 2:** Descriptive statistics for the imitation effect in the automatic imitation task.

	Median (Ms)	Range (Ms)
**Partner IME**		
Synchronous	18	− 50 to 120
Non-synchronous	− 8	− 50 to 120
**Experimenter IME**		
Synchronous	2	− 50 to 120
Non-synchronous	− 6	− 50 to 120

#### Preference overlap task

As an initial check that people correctly remembered which items were previously chosen, we first calculated recall scores separately for each individual (Self, Experimenter, Partner) as percentage correct (see Table 3). This confirmed that people were able to correctly recall which of the two items had been previously chosen for each of the individuals, meaning we created the desired context to interpret the below results.

**Table 3:** Mean percentage correct scores for the preference overlap task.

	Mean percentage correct
**Partner items**	
Synchronous	94.87
Non-synchronous	92.41
**Experimenter items**	
Synchronous	90.63
Non-synchronous	87.28
**Self items**	
Synchronous	95.76
Non-synchronous	95.31

We then analysed preference imitation using Mann Whitney U tests. These showed that there was no significant difference between conditions for Experimenter-Self preference imitation (U = 1.015, p = 0.310, r = 0.14). However, there was a non-significant trend (U = 277.0, p = 0.057, r = 0.25) towards greater preference overlap with the Partner (Partner-Self) amongst those who had taken part in the Synchronous condition. In order to follow up on this trend, a Bayesian Mann Whitney U test was also conducted. This showed greater support for the research hypothesis (that the scores of those in the Synchronous condition were higher than those in the Non-synchronous condition) (BF10 = 1.99), compared to the evidence for the null, (BF01 = 0.50). See Table 4 for descriptive statistics.

**Table 4:** Descriptive statistics for the Overlap scores form the Preference Overlap Task.

	Median	Range
**Experimenter—self imitation scores**		
Synchronous	35.94	0.0–62.50
Non-synchronous	27.68	6.25–68.75
**Partner—self imitation scores**		
Synchronous	35.94	12.50–62.50
Non-synchronous	27.78	6.25–62.50

Finally, we assessed blur scores. Following the Synchronous compared to the Non-synchronous movement task, there was less blurring for Self-Partner (U = 557.5, p = 0.003, r = 0.39) and Self-Experimenter (U = 549.5, p = 0.006, r = 0.37), but not Experimenter-Partner (U = 490.5, p = 0.093, r = 0.22). A Bayesian Mann Whitney U test showed that the research hypothesis (that the Non-synchronous condition had greater blurring scores than the Synchronous condition) was less supported (BF10 = 0.11) than the null (BF01 = 8.78). See Table 5 for descriptives. This indicates that after synchronization, participants were less likely to blur the Self with other agents.

**Table 5:** Descriptive statistics for the Blurring scores form the Preference Overlap Task.

	Median	Range
**Self—partner**		
Synchronous	1.18	0–6.25
Non-synchronous	3.65	0–34.38
**Self—experimenter**		
Synchronous	1.56	0–9.38
Non-synchronous	4.38	0–28.13
**Experimenter—partner**		
Synchronous	2.76	0–12.5
Non-synchronous	5.63	0–31.25

## Discussion

Participants showed a stronger tendency to automatically imitate actions of a co-actor with whom they had performed movements in synchrony, compared to a co-actor with whom they had not acted in synchrony, while no such difference was present for automatic imitation of the Experimenter. There was also a trend towards greater preference overlap of Partner’s post-synchronous vs non-synchronous movements, although this was not significant at the 0.05 level. The Bayesian analysis showed some support for the research over the null hypothesis, while no such difference was observed for the Experimenter. This suggests that acting in synchrony with someone may increase the tendency to imitate them, both in terms of motor imitation in the AIT, and, to a lesser extent, in terms of preference imitation.

Importantly, greater affiliation and imitation post synchronisation was only seen toward the co-actor, and not towards the experimenter who was present but not involved in the coordination. This provides further evidence that the consequences of acting in synchrony do not generalise beyond the co-actor, as some have suggested^11,12^. This finding further supports what is now the majority of work in the literature showing that the pro-social consequences of synchrony do not generalise beyond co-actors to observers^13,15–17^. Our findings add to the growing body of work which does not support the positive affect / generalised prosociality account of coordination’s social consequences^7,8,13,15–17^.

In relation to source memory, blurring scores suggested rather than memories for the self and co-actor becoming blurred, participants actually showed less blurring for the self and their co-actor following synchronous movement performance. This suggests that while synchrony may increase the tendency to imitate a co-actor, it does not necessarily blur the lines between self and other but rather improves source memories for the self. This does not support the self-other overlap account that has been suggested to account for synchrony’s social consequences^18^. It could be that an increased tendency to think about the partner's mental states following synchronous coordination^52^ helps to keep self and other apart in source memory.

Previous work on coordination's effects on memory has produced mixed findings. Research^28^ suggests that the self-reference effect (better memories for items to do with the self than another), disappeared after acting in synchrony. That is, following synchronous coordination individuals were just as good at recalling items that were co-actor-generated as those that were self-generated. Other work suggests that synchronous coordination may lead to more distinguishable or accurate source memories with respect to both the self and co-actor^29,30^. For example, following dancing in time together, individuals could better pick out co-actors' faces and attire from line ups^30^. Thus, it appears that coordination may enhance memory in general and specifically memory for the self and one's co-actors. However, our findings provide evidence that while synchronous coordination may enhance memories for the self, it does not lead to source confusion between the self and a co-actor.

In summary, Study 1 showed that following synchronous movement participants reported increased affiliation, had a tendency to imitate each other's actions and preferences, and had better source memory for the self. The coordination task utilised here involved participants not only moving at the same time as each other but also having an explicit shared goal to coordinate, that is, they were working together to achieve in-phase coordination. Coordination can vary from task to task, and one such distinguishing factor is the presence or absence of shared goals. When playing music or singing together, co-actors share a common interdependent goal, while two people walking side by side, who naturally drift into an in-phase pattern, don't share this explicit goal to coordinate. While both examples involve motoric coordination, only the first involves a shared goal to do so^53^. Some research suggests that this shared goal may play a crucial role in explaining the social consequences of coordination^5,54^. To explore this further, Study 2 replicated Study 1 replacing the intentional synchronisation task with an incidental one, where participants were asked to move in time to separate metronomes, creating either incidental synchronous or asynchronous movements.

## Study 2

### Method

Fifty-six new participants were recruited from the Central European University participant pool (34 females, 21 males, 1 other M_age_ = 26.71 yr, SD_age_ = 6.04). All participants were right-handed and naive to the aims of the study. No participants who took part in Study 1 took part in Study 2. The experiment was approved by the (EPKEB) United Ethical Review Board for Research in Psychology and took approximately 1 h to complete.

The design, procedure and materials were identical to Study 1 except for the movement task. Here, participants in each of the two conditions were given identical instructions. They were asked to hold the slider with both hands and move it from side to side, using the full range of motion without hitting the endpoints. They were asked to move their slider at a steady, continuous pace to arrive at each endpoint in time with a beep played to them for the entirety of the task through headphones. In the Synchronous Movement condition, each person heard the same metronome (37 BPM), while in the Non-synchronous Movement condition, each person heard a different metronome (32 vs 50 BPM). No occluder was used, and metronomes were played to participants through headphones. The movement task had the same number and length of trials as Study 1.

## Results

Participants in the Synchronous condition did coordinate their movements more tightly than those in the Non-synchronous condition, for trial 1 (U = 752.0, p < 0.001, r = 0.79) and trial 2 (U = 748.0, p < 0.001, r = 0.78), see Table 6 for descriptive statistics. This confirmed that the task had created the desired difference in movement synchrony for us to interpret the below results.

**Table 6:** Descriptive statistics for the coordination task.

	Median	Range
**Trial 1**		
Synchronous	3.36	1.94–12.34
Non-synchronous	25.75	10.34–29.85
**Trial 2**		
Synchronous	3.18	2.13–11.97
Non-synchronous	25.73	10.25–30.29

Scale analysis confirmed that the five affiliation items could be reliably combined into a single measure (Cronbach's alpha = 0.905). An independent-samples Mann Whitney U test showed that the difference in affiliation change scores between those in the Synchronous (Mdn = 2.4 range =  − 12.4 to 39.4) and Non-synchronous condition (Mdn = 4.6, range =  − 20.8 to 23.4) was not significant (u = 319.0, p = 0.232, r = 0.156).

For the automatic imitation task, there was no significant difference in automatic imitation of the Partner's hand between the Synchronous (M =  − 7, SD = 29) and Non-synchronous (M =  − 107, SD = 30) conditions, t(54) = 0.446, p = 0.657, d = 0.12). Mean RTs and error rates reported separately for in/congruent trials can be found in the supplementary materials.

Similarly, for the preference overlap task, recall scores again confirmed that people were able to correctly recall which of the two items had been previously chosen for each of the individuals (see Table 7 for descriptive statistics).

**Table 7:** Mean percentage correct scores for the preference overlap task.

	Mean percentage correct
**Partner items**	
Synchronous	95.31
Non-synchronous	93.97
**Experimenter items**	
Synchronous	90.85
Non-synchronous	87.72
**Self items**	
Synchronous	94.87
Non-synchronous	97.99

There was no significant difference between conditions for either the Self-Partner (U = 300.0, p = 0.128, r = 0.20) or the Self-Experimenter (U = 779.0, p = 0.75, r = 0.04) preference overlap scores. There were also no significant differences in blurring for either the Self-Partner (U = 442.5, p = 0.360, r = 0.12) or the Self-Experimenter (U = 3777.0, p = 0.784, r = 0.04) scores. See Table 8 for descriptive and inferential statistics.

**Table 8:** Descriptive and inferential statistics for the overlap and blurring scores form the preference overlap task.

	Median	Range
**Overlap scores**		
*Partner—self*		
Synchronous	37.5	6.25–75.0
Non-synchronous	31.25	6.25–75.0
*Experimenter—self*		
Synchronous	28.57	0.0–68.75
Non-synchronous	29.38	6.25–62.5
**Blurring scores**		
*Self—partner*		
Synchronous	1.43	0–15.63
Non-synchronous	2.04	0–12.50
*Self—experimenter*		
Synchronous	1.79	0–31.25
Non-synchronous	1.70	0–21.88

## Discussion

Study 2 suggests that incidental synchronisation does not have the same effects on affiliation, on the tendency to imitate actions or on memory as intentional synchronisation does. In line with previous work looking at synchrony's effects on cooperation^5^, the results reported here suggest that a shared goal may be a crucial part of the process by which synchronous coordination affects social consequences concerning affiliation, imitation and memory. Reddish^5^ suggested that synchrony's ability to foster cooperation might be dependent upon co-actors having a group goal to coordinate, which is also believed to be one of the ways in which individuals come to see each other as common group members^55^. This may, in part, explain some of the mixed findings in the literature in regards to the effects of coordination on affiliation and overlap. As the literature is awash with different types of coordination tasks, such as chanting, singing, dancing, tapping, drumming, rowing, stepping, walking, tapping (see^8^ for a review) studies differ on many fronts including the presence of shared goals. That is, coordination tasks within the literature vary with regard to whether individuals possess a shared intention to coordinate, or whether they simply end up doing so when following synchronised metronomes. Future work in this area should pay closer attention to the types of coordination task employed, particularly whether they involve shared goals to coordinate.

## General discussion

Across two studies, we examined the effect of intentional (goal-directed) and incidental (metronome following) synchronisation of movements on affiliation, automatic imitation tendencies, preference overlap and source memory. The results of Study 1 showed that post intentional synchrony individuals reported greater affiliation towards their Partner, showed a stronger tendency to imitate actions of the Partner's hand (but not the Experimenter's hand), had slightly greater preference sharing with Partner (but not the Experimenter) and had less memory blurring for Self vs Partner/Experimenter items but not Experimenter/Partner items. Study 2 involving incidental coordination did not replicate any of these findings. When coordination was incidental rather than intentional, there were no differences between the Synchronous and Non-synchronous conditions across the board.

The pattern of findings reported here suggests that a shared goal may be crucial for synchronous movement performance to lead to greater affiliation and the tendency to imitate the actions of one's co-actor. However, it is important to note that in Study 1 an occluder was used in the control condition in order to keep participants from shifting to a coordinated state^4^. In terms of automatic action imitation (as measured by the IAT) this may result in a potential confounding factor. It may be that the greater automatic imitation seen in the synchronous over the non-synchronous condition was reliant on participants seeing the co-actor's hand during the coordination task. To rule out this confound, we performed an additional analysis comparing the Imitation Effect scores following intentional vs incidental coordination. This exploratory analysis can be found in the supplementary materials. This analysis suggested that the greater imitation seen following intentional coordination is not simply due to the participants being able to see each other's hand during the coordination task, but due to the type of coordination task itself. If observation of the co-actor's hand during the movement task was sufficient for enhancing the tendency to imitate their actions, Study 2 should have revealed a stronger tendency to imitate the co-actor's hand than the experimenter's hand. This was not the case. Still, a potential role of attention cannot be conclusively ruled out, and this should be addressed in future work.

Our results showed that synchronous movement, when intentional, not only increases affiliation and the tendency to imitate a partner but may also strengthen source memories for the self. Together, this suggests that intentional synchronisation does not lead to self-other overlap resulting in greater source confusion with the Partner; instead, we actually saw better source memory accuracy for the self. Changes in group categorisation induced by synchronisation may provide a more suitable explanation. That is, rather than people viewing their co-actor as an extension of themselves, they may come to view them as part of the same social group as themselves. Intentional synchronisation may lead individuals to group the Partner with the self, leading to stronger source memory encoding and a greater implicit desire to imitate the partner.

Critical to this interpretation is the finding that incidental coordination did not lead to the same effects. This suggests that individuals must conceive of the coordination as a joint endeavour with mutually desired movement goals. Indeed, shared goals are a factor thought to be crucial for categorising individuals as members of the same social group^8^. Indeed people have been shown to be likely to rate moving agents as more entitative (a cohesive unit rather than separate individuals), based not only on how coordinated their movements were, but also whether they believed they were coordinating their movements freely or had been instructed to do so^56^.

One limitation of the current study is that it did not directly compare incidental and intentional coordination. Future work should disentangle the relation between synchronisation and joint goals using 2 × 2 designs, manipulating the presence of synchrony and the presence of shared coordination goals, followed by similar measures assessing affiliation, imitation, overlap and memory. It may be that the presence of a joint movement goal is in and of itself sufficient for pro-social effects to appear, however, the addition of synchrony to a joint movement goal may add additional strength to these effects. A further limitation is that, like most studies on the impact of in-phase synchrony, the present study compared the performance of synchronous movements with non-synchronous movements, but did not investigate the effects that other forms of coordination, like anti-phase coordination or turn-taking, have on affiliation, automatic imitation, and memory. Finally, it is debated to what extent the AIT measures imitative tendencies per se or more domain-general mechanisms unrelated to imitative processes (for a review of this issue see^57^). Future work should try to extend the current findings to other implicit tasks that involve well-specified social processes.

In conclusion, a clear pattern of findings emerged whereby intentional synchronisation led to greater affiliation and automatic imitation of a Partner than either incidental coordination or uncoordinated movements. No evidence of source memory confusion between Self and Partner was observed. This work advances our understanding of how coordination can affect affiliation, imitation and memory of our co-actors and suggests that shared goals play a key role.

## Supplementary Information

Supplementary Information.
