# Supplementary material for: Intentional synchronisation affects automatic imitation and source memory
Source: Sci Rep. 2021 Jan 12;11:573. doi: 10.1038/s41598-020-79796-9 (PMC7804244; doi:10.1038/s41598-020-79796-9)
Supplement: Supplementary file 1 — Supplementary Information. [file 41598_2020_79796_MOESM1_ESM.docx]

**SUPPLEMENTARY MATERIALS**

***Exploratory Analysis***

An additional independent samples t-test was conducted comparing IME scores of the Synchronous condition of Study 1 and 2 to rule out that any differences in imitation effect seen in Study 1 could be solely due to the use of the occluder in the Non-synchronous condition. Parametric t-tests were used in lieu of non-parametric Mann Whitney U tests as while the data of the Non-synchronous condition differed significantly from normality, the Synchronous condition did not. Intentional synchronization (Study 1) resulted in significantly higher IME scores than incidental synchronization (study 2) (T(52)=2.65, p=.011, d= 0.72). This confirmed that IME findings of Study 1 were not merely due to participants being able to view each other's hand in the Synchronous version of the movement task.

***Additional Descriptives***

Mean RTs (in ms) and error rates in the AIT for the Partner and Experimenter in Study 1 and 2 reported separately for in/congruent trials in the table below.

|  |  |  |  | RT | SD | Error rate | SD |
| --- | --- | --- | --- | --- | --- | --- | --- |
| Study 1 | Partner | Congruent trials | Synchronous | 375 | .048 | .048 | .061 |
|  |  |  | Non-synchronous | 389 | .070 | .060 | .080 |
|  |  | Incongruent trials | Synchronous | 392 | .064 | .103 | .167 |
|  |  |  | Non-synchronous | 399 | .067 | .086 | .107 |
|  | Experimenter | Congruent trials | Synchronous | 378 | .048 | .036 | .034 |
|  |  |  | Non-synchronous | 383 | .056 | .063 | .067 |
|  |  | Incongruent trials | Synchronous | 382 | .044 | .071 | .146 |
|  |  |  | Non-synchronous | 375 | .050 | .085 | .127 |

| Study 2 | Partner | Congruent trials | Synchronous | 396 | .069 | .036 | .042 |
| --- | --- | --- | --- | --- | --- | --- | --- |
|  |  |  | Non-synchronous | 388 | .063 | .054 | .065 |
|  |  | Incongruent trials | Synchronous | 404 | .080 | .073 | .100 |
|  |  |  | Non-synchronous | 399 | .060 | .052 | .065 |
|  | Experimenter | Congruent trials | Synchronous | 392 | .046 | .045 | .059 |
|  |  |  | Non-synchronous | 373 | .052 | .045 | .045 |
|  |  | Incongruent trials | Synchronous | 393 | .061 | .049 | .063 |
|  |  |  | Non-synchronous | 384 | .058 | .070 | .079 |
